# TZAP plays an inhibitory role in the self-renewal of porcine mesenchymal stromal cells and is implicated the regulation of premature senescence via the p53 pathway

**DOI:** 10.1186/s12967-019-1820-8

**Published:** 2019-03-07

**Authors:** Ya-nan Bie, Peng Gu, Yu-ting Chen, Xiao-xu Zhou, Yu-guang Tian, Qin Yang, Hai-yan Li, Xia Lin, Yan-hong Guan, Tao-yan Lin, Xun Lu, Hong-fen Shen, Ting-xiao Fang, Yu-min Liu, Dong Xiao, Wei-Wang Gu

**Affiliations:** 1 Institute of Comparative Medicine & Laboratory Animal Center, Southern Medical University, Guangzhou, China; 2 Songshan Lake Pearl Laboratory Animal Sci & Tech. Co., Ltd., Dongguan, China; 3 Department of Toxicology, School of Public Health, Southern Medical University, Guangzhou, China; 4 Guangdong Provincial Key Laboratory of Cancer Immunotherapy Research and Guangzhou Key Laboratory of Tumor Immunology Research, Cancer Research Institute, Southern Medical University, Guangzhou, China; 5 Department of Pharmacy, Nanfang Hospital, Southern Medical University, Guangzhou, China

**Keywords:** TZAP, pMSCs, Senescence, Tibet minipigs, CRISPR/Cas9, p53

## Abstract

**Background:**

Mesenchymal stromal cells (MSCs) were originally characterized by the ability to differentiate into different mesenchymal lineages in vitro, and their immunomodulatory and trophic functions have recently aroused significant interest in the application of MSCs in cell-based regenerative medicine. However, a major problem in clinical practice is the replicative senescence of MSCs, which limits the cell proliferation potential of MSCs after large-scale expansion. Telomeric zinc finger-associated protein (TZAP), a novel specific telomere-binding protein, was recently found to stimulate telomere trimming and prevent excessive telomere elongation. The aim of this study was to elucidate the role of TZAP in regulating MSCs senescence, differentiation and proliferation.

**Method:**

Primary porcine mesenchymal stromal cells (pMSCs) were isolated from the bone marrow of Tibet minipigs by a noninvasive method in combination with frequent medium changes (FMCs). The deterioration of the pMSCs’ proliferation capacity and their resultant entry into senescence were analyzed by using CCK8 and EdU incorporation assays, SA-β-gal staining and comparisons of the expression levels of cellular senescence markers (p16INK14 and p21) in pMSC cell lines with TZAP overexpression or knockout. The effects of TZAP overexpression or knockout on the differentiation potential of pMSCs were assessed by alizarin red S staining after osteogenic induction or by oil red O staining after adipogenic induction. The effect of TZAP overexpression and the involvement of the p53 signaling pathway were evaluated by detecting changes in ARF, MDM2, P53 and P21 protein levels in pMSCs.

**Results:**

TZAP levels were significantly elevated in late-passage pMSCs compared to those in early-passage pMSCs. We also observed significantly increased levels of the senescence markers p16INK4A and p21. Overexpression of TZAP reduced the differentiation potential of the cells, leading to premature senescence in early-passage pMSCs, while knockout of TZAP led to the opposite phenotype in late-passage pMSCs. Furthermore, overexpression of TZAP activated the P53 pathway (ARF-MDM2-P53-P21WAF/CDKN1A) in vitro. TZAP also downregulated the expression levels of PPARγ and Cebpα, two key modulators of adipogenesis.

**Conclusions:**

This study demonstrates that the level of TZAP is closely related to differentiation potential in pMSCs and affects cellular senescence outcomes via the p53 pathway. Therefore, attenuation of intracellular TZAP levels could be a new strategy for improving the efficiency of pMSCs in cell therapy and tissue engineering applications.

**Electronic supplementary material:**

The online version of this article (10.1186/s12967-019-1820-8) contains supplementary material, which is available to authorized users.

## Background

Mesenchymal stromal cells (MSCs) were initially identified and characterized by their multilineage differentiation potential in vitro and were plastic-adherent nonhematopoietic cells [1–3], which contain the ability to form fibroblastoid colonies (CFU-F) [4]. However, the hallmark stem cell characteristics of self-renewal and differentiation have not been clearly demonstrated for these cells in vivo [5]. Moreover, MSCs do not reconstitute diseased or missing tissue but home to sites of disease and secrete bioactive factors [6] that are immunomodulatory and trophic [7] (regenerative) to facilitate tissue repair. There is extensive controversy concerning the naming of exogenously supplied MSCs as mesenchymal stromal cells or other terms, including medicinal signaling cells, which aim to reflect the in vivo therapeutic function of these cells more accurately [8]. Nevertheless, the potent therapeutic benefit of MSCs has been pursued in cell-based interventions in regenerative medicine and tissue engineering [9]. These cells hold great promise for the treatment of various tissue injuries in preclinical and clinical settings, including the regulation of angiogenesis and growth, the differentiation of local progenitor cells, the prevention of fibrosis and apoptosis, and the modulation of immune responses [10–12].

However, after 20–40 rounds of cell division in vitro, MSCs exhibit replicative senescence and stop proliferating. The senescence phenotype of MSCs is characterized by an enlarged size, a “fried egg morphology”, growth arrest in the G1 phase of the cell cycle, increased expression of senescence-associated β-galactosidase (SA-β-gal) and attenuated expression of specific markers [13]. Furthermore, the multilineage differentiation potential of MSCs is impaired after long-term culture, as the adipogenic potential of MSCs declines with consecutive cell passages under normal culture conditions [14, 15]. Unfortunately, the progressive loss of proliferation ability and the reduction of differentiation potential negatively impact large-scale cell expansion in vitro, which is required for cellular therapy using MSCs. Thus, the feasibility of clinical therapy using MSCs continues to be a challenge. Therefore, it is necessary to overcome the premature senescence of MSCs to enable their use in cellular therapy.

The phenomenon of replicative senescence is the so-called “Hayflick limit”, which is closely associated with the continuous erosion of telomeres due to the accumulation of DNA damage [16]. It has been observed that telomeres are shortened by an average of 17 bp/year during aging in human fibroblasts [17]. Moreover, telomeric DNA undergoes gradual erosion after continued population doublings (PDs), at approximately 50–100 bp/PD [18]. When telomeres are shortened to 5.8–10.5 kb [19] and accompanied by increasing DNA damage [20], MSCs ultimately cease growth. Previous studies have demonstrated that telomere shortening is required for senescence in MSCs [21], and DNA damage is localized to telomeric DNA in senescent MSCs [22].

Recently, Li et al. reported that telomeric zinc finger-associated protein (TZAP), known as Krüppel-like zinc finger protein (ZBTB48), is a new telomere-binding protein involved in the initiation of the trimming process. Those authors demonstrated that TZAP binds directly to TTAGGG telomere repeats, functionally replacing the DNA binding domain of telomeric repeat–binding factor 2 (TRF2), which acts as the telomere capping factor. Interestingly, overexpression of TZAP in cells’ alternative lengthening of telomeres (ALT) triggers complete deletion of telomeric DNA and renders the chromosome ends fully exposed to degradation and recombination, leading to severe genome instability; in contrast, silencing of TZAP in mouse embryonic stem cells caused telomere re-elongation and decreased extrachromosomal telomeric DNA. Overall, these results suggest that TZAP inhibits excessive telomere elongation through inducing the initiation of the trimming process [23].

The induction of replicative senescence by critically short telomeres is frequently linked to the CDKN2A locus encoding the p16 and alternative open reading frame (ARF) genes, whose expression is sufficient to activate tumor suppressor networks. The expression of p16INK4a and ARF is normally very low in young tissues but increases markedly in almost all rodent tissues with aging [24]. ARF is a tumor suppressor protein that is upregulated during replicative senescence. ARF positively regulates p53 stability through inactivating the p53 proteasomal degrading E3 ubiquitin ligase MDM2. ARF forms a complex with MDM2 and sequesters it away from p53, thereby preventing negative feedback regulation of p53 by Mdm2 and stabilizing p53 in the nucleoplasm, resulting in cell cycle arrest in the G1 and G2 phases or apoptosis [25, 26]. In addition, DNA damage may also reduce ARF expression by inducing its degradation [27, 28].

However, the role of TZAP in the regulation of telomere homeostasis and cellular senescence in MSCs remains largely unknown. Since overexpression of TZAP prevents excessive telomere elongation and induces activation of the trimming process [23], we hypothesize that TZAP may modulate cellular replication capacity through regulating telomere lengthening, which may reverse or slow age-related degeneration. This investigation will increase our understanding of the replicative senescence state of MSCs and may help establish a new mode of therapy for tissue repair in regenerative medicine and for the maintenance of physiological function in aging tissues.

In this study, overexpression and knockout of TZAP were employed in Tibet minipig MSCs by lentiviral-mediated transduction and CRISPR/Cas9 genome editing to explore the role of TZAP in senescence. Tibet minipig MSCs were chosen because they are the most attractive animal model for preclinical studies and share considerable physiological compatibility with humans, as exhibited in studies of ultraviolet radiation, cosmetic identification, frostbite, burns, etc. [29–32]. Our results indicated that the level of TZAP was increased in late-passage MSCs and that TZAP may regulate senescence in pMSCs. Thus, TZAP may be a new target for genetic modification to overcome the senescence of MSCs during large-scale ex vivo expansion.

## Method

### Animals

Tibet minipigs at the age of 6 months were obtained from the Institute of Comparative Medicine and Laboratory Animal Center of Southern Medical University (Guangzhou, Guangdong, China). The animals were housed under standard laboratory conditions and given food and water ad libitum by Southern Medical University Laboratory Animal Center. All experiments using experimental minipigs were approved by the Animal Care and Use Committee of the Animal Center of Southern Medical University (Animal Welfare Assurance L2016088).

### Isolation and cultivation of bone marrow-derived MSCs from Tibet minipigs

Primary pMSCs were isolated from the bone marrow of Tibet minipigs as previously described by Lee et al. [33] and Soleimani et al. [34]. All procedures were performed under full anesthesia and aseptic conditions. After sterilization, bone marrow was extracted from the iliac crest bone by a bone marrow extractor in a noninvasive manner and was carefully pipetted onto Ficoll-Paque solution (GE Healthcare, Piscataway, NJ, USA) at a ratio of 4:3 without mixing. The mixture was centrifuged at 500 rpm for 10 min at room temperature, and pMSCs were collected from the interface layer.

Cells were plated into a series of 25 cm^2^ flasks at a density of 7.5 × 10^5^ cells per flask in Dulbecco’s modified Eagle’s medium (DMEM; Corning, NY, USA) supplemented with 10% fetal bovine serum (FBS; Invitrogen, Paisley, UK), 10 ng/ml basic fibroblast growth factor (bFGF; Sigma-Aldrich, St. Louis, MO), 1.0% Glutamax (Gibco, Shanghai, China), and 1.0% penicillin–streptomycin (Gibco). Half of the medium was replaced with fresh medium, and non-adherent cells were carefully removed after 4 h. Thereafter, half of the medium was changed every 8 h for up to 3 days after the initial culture. The adherent fibroblast-like and spindle-shaped cells were washed with phosphate-buffered saline (PBS) and replaced with fresh medium every 2 days.

### Flow cytometric analysis

The MSC-like cells were harvested by trypsinization (Gibco) and incubated with 1% bovine serum albumin (BSA) for 1 h at 4 °C to block nonspecific Fc-mediated interactions, then incubated in the dark at 4 °C for 30 min with 400 μl of CD45-FITC (BD Biosciences, 340664, Franklin Lakes, NJ), CD34-APC (BD, 555824), CD105-FITC (BD, 561443), CD73-PE (BD, 561258), and CD90-APC (BD, 559869) antibodies. The cells were stained with PE- or FITC-labeled IgG as an isotype control. The cells were evaluated by a FACSCalibur flow cytometer (Becton–Dickinson, San Jose, CA) and analyzed with FlowJo software (Tree Star Inc., Ashland, Oregon, USA). The percentage of stained cells was calculated relative to the isotype control.

### Differentiation assays

In vitro differentiation ability was examined in mesenchymal lineages (osteocytes, adipocytes, and chondrocytes). For osteogenesis, pMSCs were seeded into 6-well plates at a density of 2 × 10^6^ cells per well. When the cells reached 80% confluence, the cell culture medium was supplemented with osteogenic differentiation medium (Cyagen Biosciences, Guangzhou, China) containing 10% FBS, 10 mM β-glycerophosphate, 0.1 mM ascorbic acid, and 10 nM dexamethasone in DMEM. The medium was replaced every 3 days for 1 week. Then, the cells were stained with 1% alizarin red S and analyzed under an inverted microscope (Nikon Eclipse Ti-S inverted microscope, USA).

For adipogenesis, pMSCs were seeded into 6-well plates at a density of 2 × 10^6^ cells per well. The cells were treated with adipogenic differentiation induction medium (Cyagen) containing 10 mg/ml insulin, 100 mM indomethacin, 500 mM 3-isobutyl-1-methyl xanthine, and 1 mM dexamethasone in DMEM for 3 days, and the medium was replaced with adipogenic differentiation maintenance medium (Cyagen) containing 10% FBS and 10 mg/ml insulin in DMEM for another 24 h. After 5 induction-maintenance cycles, the culture conditions were changed to adipogenic differentiation maintenance medium with replacement every 3 days. After 7 days, the cells were stained with oil red O according to standard protocols (Cyagen) and analyzed under an inverted microscope (Nikon).

For chondrogenesis, pMSCs were supplemented with chondrogenic differentiation medium (Cyagen) containing 10 ng/ml transforming growth factor (TGF)-β3, 40 mg/ml proline, 100 mg/ml pyruvate, 10^−7^ M dexamethasone, a 1:100 dilution of ITS + Premix, and 50 mg/ml ascorbate-2-phosphate, which was replaced every 3 days. After 2–3 weeks of induction, the cells were stained with Alcian blue and subjected to histological analysis.

### Plasmids

Human cDNA for Zbtb48 was purchased from Vigene Biosciences (Vigene Biosciences, NM0_005341, Shandong, China) and cloned into the pCDH-GFP-puro lentiviral vector to generate the pCDH-TZAP-GFP-puro recombination plasmid. The other plasmids used in this study were lentiCRISPRV2 (Addgene, Plasmid 52961), psPAX2 (Addgene, Plasmid 12260) and pMD2G (Addgene, Plasmid 12259).

### Lentivirus-mediated transduction

pCDH-TZAP-GFP-puro or the pCDH-GFP-puro control plasmid was transfected into HEK293T cells with the packaging plasmids psPAX2 and pMD2G using Lipofectamine 2000 (Invitrogen). The culture supernatant was collected after 72 h, and the harvested lentiviruses were used to infect target pMSC cells. For lentiviral infection, pMSCs were seeded into 6-well plates and grown to 80% confluence, followed by incubation with the LV-TZAP or LV-GFP virus for 24 h and selection with 2.5 μg/ml puromycin for 3 days.

### CRISPR/Cas9 targeting

TZAP knockout cells were generated by using lentiviral CRISPR/Cas9 to facilitate specific genome editing in pMSCs. The target sequence of the TZAP sgRNA was designed through the CRISPR Design Tool (http://crispor.tefor.net/). Targeting of TZAP in pMSCs was carried out with the following sgRNA: TGCGATGCCACCTTGGACGT (Exon 2). The synthetic gRNA oligos were annealed following a standard protocol, cloned into the vector ‘LentiCRISPR v2’ at the BsmBI restriction site and confirmed by sequencing [35]. Then, the recombination plasmid was cotransfected into HEK293T cells with the packaging plasmids psPAX2 and pMD2G to produce lentivirus. The lentivirus was used to infect the target cells at a multiplicity of infection (MOI) of 10. LentiCRISPRv2 without gRNA was used as a control. Antibiotic selection was performed for 3 days after transduction.

### Mutation analysis

Primers were designed for the pig TZAP gene. Genomic DNA was isolated using a TIANamp Genomic DNA Kit (Tiangen, Beijing, China) according to the manufacturer’s instructions. The primers used to detect TZAP mutations were as follows: TZAP-F: 5′-AGGCTTTCTCTTGCTACC-3′ and TZAP-R: 5′-CAATCCTCAGGCTCCTTG-3′. The PCR products were then gel-purified and sequenced to confirm successful gene editing. The efficiency and spectra of the mutations were analyzed by TIDE software [36].

### Cell counting kit-8 (CCK8) assay

To quantitatively determine cell proliferation, pMSCs were seeded into 96-well tissue culture plates at a density of 2500 cells per well in complete culture medium. Three replicates were set up for the test. The number of cells was estimated using a CCK8 assay (Bestbio, Shanghai, China) at each time point (24, 48, 72, 96, and 120 h) according to the manufacturer’s instructions. Then, 10 μl of CCK8 was added to each well and incubated for another 2 h. Optical density values (OD) were measured at a 450 nm wavelength on a microplate reader (Molecular Devices, Sunnyvale, CA, USA).

### 5-Ethynyl-2′-deoxyuridine cell proliferation assay (EdU assay)

The assay was performed by using a Cell-Light™ EdU Apollo 567 In Vitro Imaging Kit (EdU; Guangzhou RiboBio, Guangzhou, China) as described previously [37]. Briefly, 5000 pMSCs were plated into 96-well tissue culture plates for 48 h, and the cells were exposed to EdU at a concentration of 50 μM and incubated for 2 h, followed by fixation with 4% formaldehyde for 30 min.

After fixation, the cells were stained with the staining mixture for 30 min and then washed twice with PBS/0.5% Triton X-100. Finally, the cells were counterstained with Hoechst 33342 and imaged by fluorescence microscopy (Nikon Eclipse Ti-S inverted microscope, USA).

### SA-β-gal staining

Staining of SA-β-gal was performed by using the Senescence β-Galactosidase Staining Kit (Cell Signaling Technology, Danvers, MA) according to the manufacturer’s specifications. pMSCs were plated into a 96-well plate and fixed with 1× fixative solution. After washing with ice-cold PBS, freshly prepared β-galactosidase staining solution was added to each well. The plates were sealed and incubated overnight at 37 °C without CO_2_. The cells were analyzed under a light microscope. The results were expressed as the percentage of cells positive for blue staining in each well.

### Western blot analysis

The cell lysates were separated by 10–15% sodium dodecyl sulphate–polyacrylamide gel electrophoresis (SDS-PAGE) and electrophoretically transferred to a PVDF membrane (Millipore, Billerica, MA). The membrane was incubated with primary antibodies, including rabbit anti-ZBTB48 (Abcam, ab50588, Cambridge, United Kingdom), rabbit anti-GAPDH (Proteintech Co., 10494-1-AP, Wuhan, China), rabbit anti-P21 (Proteintech, 10355-1-AP), rabbit anti-P53 (Proteintech, 10442-1-AP), rabbit anti-CDKN2A/P16-INK4a (Bioss Co., bs-4592R, Beijing, China), rabbit anti-P14ARF (Bioss, bs0534R), rabbit anti-MDM2 (Bioss, bs-23748R), and rabbit anti-PPAR gamma (Bioss, bs-4590R) antibodies overnight at 4 °C, followed by incubation with an HRP-labeled goat or mouse anti-rabbit IgG for 1 h at room temperature. Specific proteins were detected by a high-sensitivity chemiluminescence imaging system (BioRad Laboratories, Hercules, CA, United States). Glyceraldehyde-3-phosphate dehydrogenase (GAPDH) was used as a control.

### Quantitative real-time PCR analysis (qRT-PCR)

Total RNA was extracted from cells using TRIzol Reagent (Takara Biochemicals, Dalian, China). cDNA was synthesized by the PrimeScript™ RT Reagent Kit (Takara) and amplified as the template by qRT-PCR with SYBR Premix Ex Taq™ (Takara) according to the manufacturer’s instructions. Relative gene expression was analyzed using the 2^−∆∆Cт^ method. The primer sequences are listed in Additional file 1: Table S1.

### Statistical analysis

In each experiment, data were acquired from three independent experiments and presented as the mean ± SEM after statistical processing. Statistical analysis was performed using SPSS 20.0 software and GraphPad 6.0 software. Comparisons were assessed by independent two-tailed Student’s t tests and one-way analysis of variance (ANOVA). The Student–Newman–Keuls multiple range test was used to determine the statistical significance of differences between means when a significant F ratio was found. *p < 0.05, **p < 0.01 and #*p* < 0.001 were considered to indicate different degrees statistical significance.

## Results

### Characterization of pMSCs in vitro

**Fig. 1:**
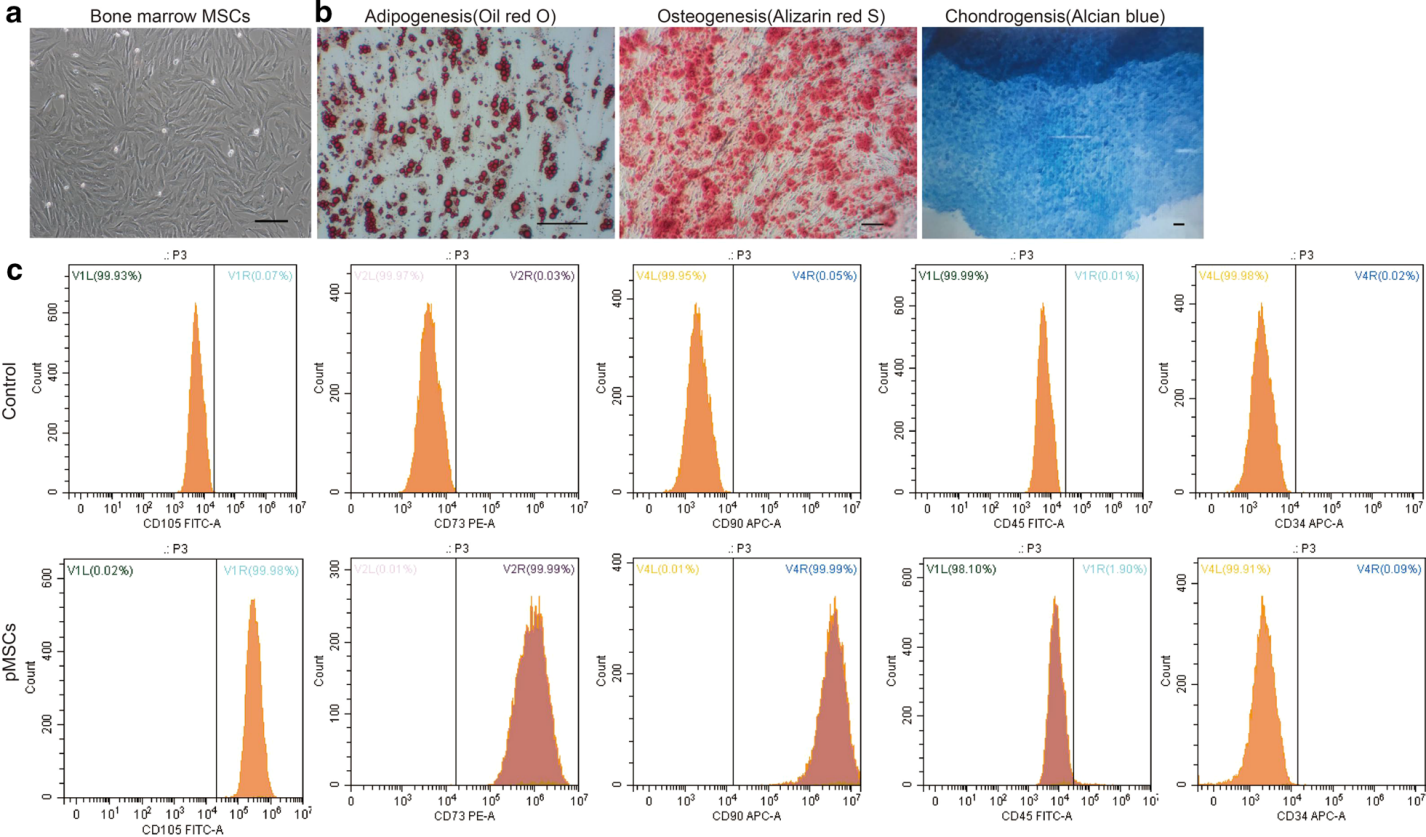
Identification of pMSCs isolated from the bone marrow of Tibet minipigs. **a** Bar = 100 μm. Fibroblast-like morphology of pMSCs isolated from the bone marrow of Tibet minipigs. **b** Bar = 100 μm. Trilineage differentiation potential of pMSCs demonstrated at P3. Oil red O, alizarin red S, and Alcian blue staining were used to determine the adipogenic, osteogenic, and chondrogenic differentiation of pMSCs, respectively. **c** FACS analysis of pMSC-specific markers on the cell surface at P3, and pMSC identity was confirmed by the detection of positive results for CD105, CD90, and CD73 and negative results for CD45 and CD34. The control cells were stained with a nonimmunoreactive isotype control antibody. Top row, images of the isotype controls. Bottom row, images of the experimental flow cytometric analyses

Primary pMSCs were isolated from the bone marrow of Tibet minipigs using a noninvasive method combined with flow cytometry (FCM), as previously described [33, 34]. All of the criteria for MSCs were fulfilled. The morphology of the pMSCs was fibroblast-like, spindle-shaped and homogeneously adherent, as exhibited in passage 2 pMSCs (Fig. 1a). Furthermore, the pMSCs were able to differentiate toward adipocytes, osteoblasts and chondrocytes (Fig. 1b). The results of FCM analysis showed that the pMSCs expressed the mesenchymal markers CD105, CD90, and CD73, while the expression of the hematopoietic cell markers CD45 and CD34 was negligible (Fig. 1c).

### TZAP expression increased during aging in pMSCs

**Fig. 2:**
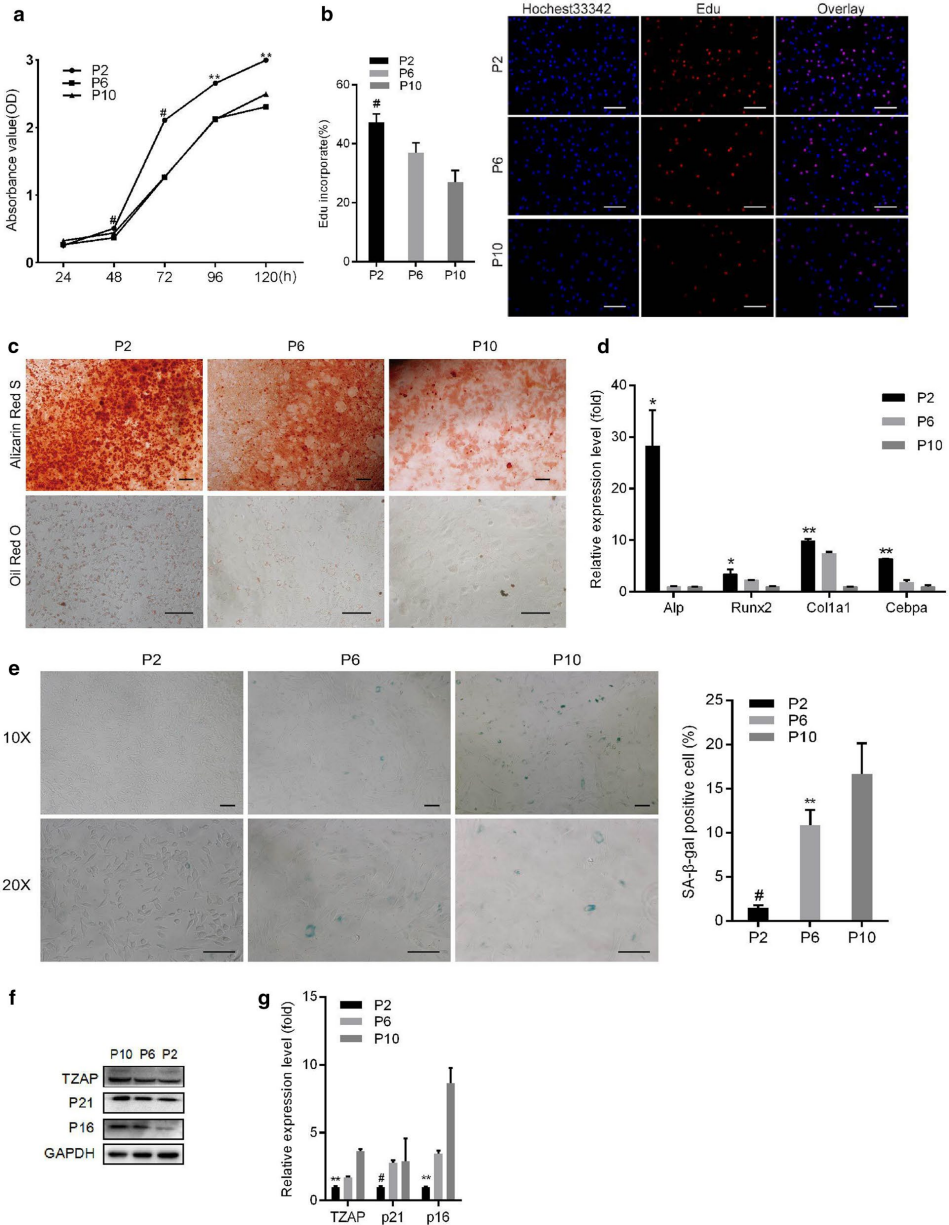
TZAP expression increased with pMSC aging (P2, P6 and P10). The rates of cell growth and EdU incorporation were assayed by a CCK8 (**a**) assay and an EdU assay (**b**), respectively. Bar = 100 μm. **c** Bar = 100 μm. The osteogenic and adipogenic potential of pMSCs at different passages was assessed by alizarin red S staining and oil red O staining, respectively, after induction of differentiation. **d** The expression of bone markers (*Alp*, *Runx2*, and *Col1a1*) and adipogenic markers (*Cebpa*) was assessed by qRT-PCR after adipogenic and osteogenic induction, respectively, in pMSCs at different passages. **e** Bar = 100 μm. Comparison of the SA-β-gal staining profiles of pMSCs at different passages. **f** Protein levels of TZAP, P16INK14 (P16) and P21 in pMSCs at different passages were compared through western blot analysis. **g** The mRNA levels of TZAP, *p16INK14* (p16) and *p21* in pMSCs at different passages were detected by qRT-PCR. All data are represented as the mean ± SEM. n = 3. *p < 0.05; **p < 0.01; ^#^*p *< 0.001, tested by one-way ANOVA

It was reported that long-term culture of MSCs induces replicative senescence, which is accompanied by repressed proliferation capacity and multipotent differentiation potential [38]. To examine the stem-like properties of expanded pMSCs, the cells were continuously cultured and evaluated during passage 2, passage 6, and passage 10 (P2, P6, and P10). We found that the cell growth rate and EdU incorporation rate were significantly reduced at late passages (P6 or P10) compared to those at early passages (P2) (Fig. 2a, b). Late-passage pMSCs showed a repressed capacity for adipogenic and osteogenic differentiation in a passage-dependent manner (Fig. 2c), which was validated by downregulation of the mRNA levels of osteoblast-specific transcripts (*Alp*, *Col1a1*, *Runx2*) and adipocyte-related markers (*Cebpa*) (Fig. 2d). In P10 pMSCs, the percentage of positive cells after staining with the senescence marker β-galactosidase (β-gal; Fig. 2e) increased from 2% to 17% (P2 to P10). These data suggest that pMSCs at early passages have better differentiation and proliferation potential than pMSCs at late passages and that pMSCs undergo senescence with an increasing number of cell passages.

To demonstrate the potential role of TZAP in the senescence of pMSCs, we assessed the expression level of TZAP and several cellular senescence markers (p16INK14 and p21). We observed that the expression of TZAP increased progressively in pMSCs during their progression from early (P2) to late passages (P6 and P10), and the upregulated expression pattern observed at late passages was consistent with the patterns observed for p16INK14 and p21 (Fig. 2f, g). These observations indicated that TZAP expression may correlate with the progression of MSC aging.

### TZAP knockout increased the proliferation capacity and differentiation potential of pMSCs

**Fig. 3:**
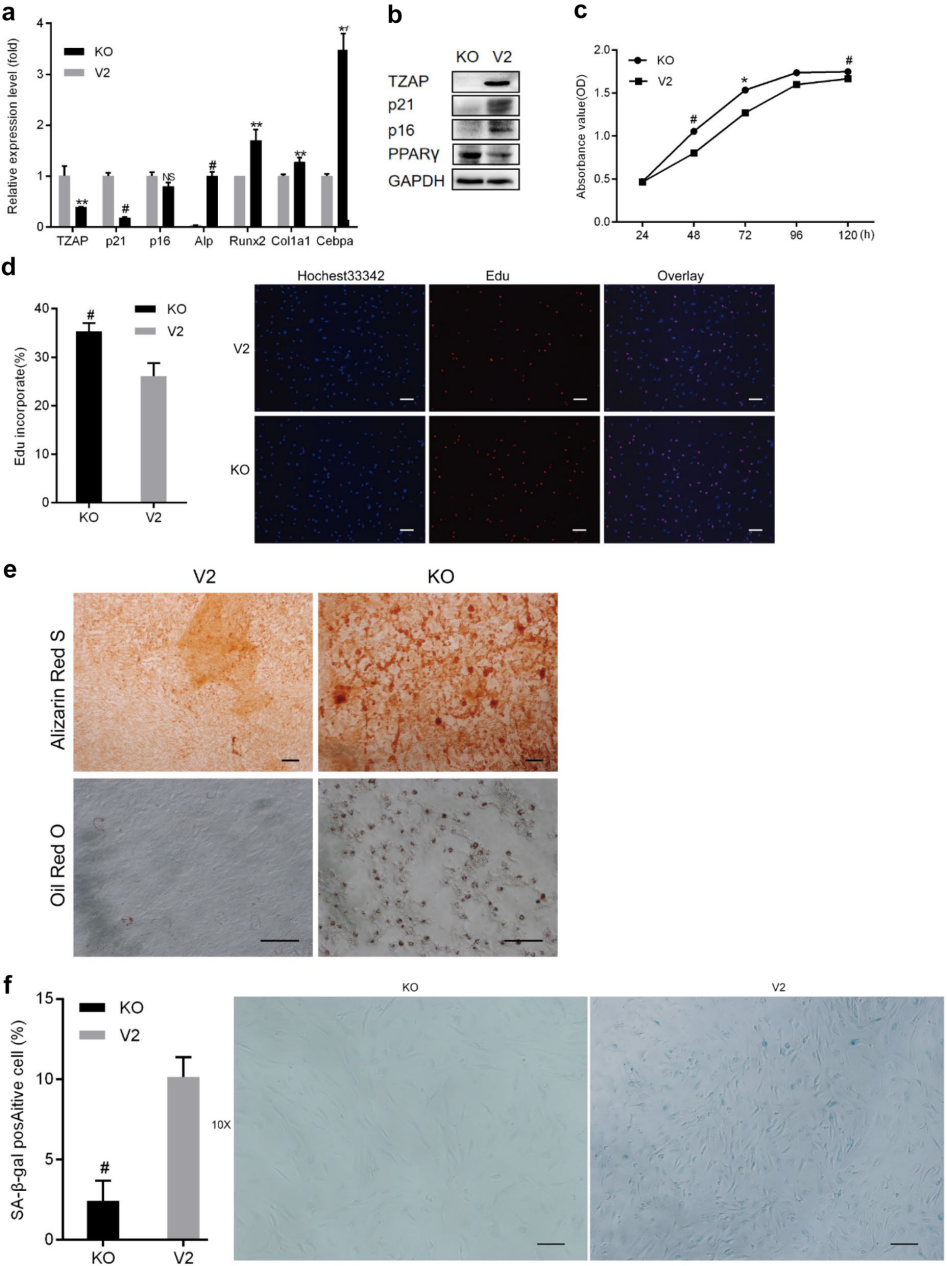
Knockout of TZAP increased the proliferation capacity and osteogenic and adipogenic differentiation potential of pMSCs. **a** Expression levels of TZAP, *p16INK14* (p16) and *p21,* adipogenic markers (*Cebpa*) and bone markers (*Alp*, *Runx2*, and *Col1a1*) were examined by qRT-PCR after TZAP knockout. **b** Protein levels of TZAP, P16INK14 (P16), P21 and PPARγ in pMSCs were compared in the control cell line (V2) and the TZAP knockout cell line (KO) through western blot analysis. **c** The cell growth rate of TZAP knockout pMSCs was assayed by a CCK8 assay. **d** Bar = 100 μm. The EdU incorporation rate of TZAP knockout pMSCs. **e** Bar = 100 μm. Differentiation of TZAP knockout pMSCs into the osteogenic and adipogenic lineages was identified using alizarin red S and oil red O staining, respectively. **f** Bar = 100 μm. Comparison of the SA-β-gal staining profile of TZAP knockout pMSCs to that of the control cell line. All data are represented as the mean ± SEM. n = 3. *p < 0.05; **p < 0.01; ^#^*p *< 0.001, tested by one-way ANOVA

To examine the roles of TZAP in pMSC proliferation and differentiation, TZAP in P10 pMSCs was knocked out by CRISPR-Cas9 genome editing. Since it was difficult to expand pMSCs from a single TZAP^−/−^ clone at low density, we used pMSCs carrying the inactivated TZAP gene, which was selected by puromycin rather than isolating a single cell clone. Compared to the control cell line, CRISPR-Cas9-mediated knockout of the TZAP gene resulted in reduced TZAP mRNA (Fig. 3a) and protein levels (Fig. 3b). Additionally, knockout of TZAP increased the rate of proliferation (Fig. 3c) and EdU incorporation (Fig. 3d).

In addition, P10 pMSCs with TZAP knockout had increased osteogenic and adipogenic differentiation potential compared to that of the control vector cell lines after culture in osteogenic induction medium or adipogenic induction medium for 1 week or 21 days, respectively (Fig. 3e). Similarly, qRT-PCR revealed that TZAP knockout increased the mRNA levels of *Alp*, *Col1a1* and *Runx2* after osteogenic induction (Fig. 3a). Adipogenic induction by oil red O staining revealed that compared to the control vector, TZAP knockout in P10 pMSCs led to a dramatically increased number of lipid-accumulating cells (Fig. 3e). Similarly, P10 pMSCs with TZAP knockout had significantly higher mRNA levels of *Cebpa* (Fig. 3a) and higher protein levels of PPARγ (Fig. 3b) than control vector cells after adipogenic induction. Taken together, these results indicate that knockout of TZAP in P10 pMSCs enhanced the proliferation capacity and differentiation potential of pMSCs.

### TZAP knockout inhibited premature senescence in pMSCs

As shown in Fig. 3b, TZAP knockout decreased the protein levels of P21 and P16, as indicated by western blotting. qRT-PCR also revealed that knockout of TZAP in pMSCs decreased the mRNA level of p21 (Fig. 3a) and the protein levels of P21 and P16INK4A (Fig. 3b) compared to those of the control vector cell lines. Moreover, SA-β-gal staining revealed that pMSCs with TZAP knockout had a reduced percentage of cells positively stained for SA-β-gal, with a value as low as nearly 2% (Fig. 3f). Taken together, these results suggest that knockout of TZAP in late-passage pMSCs helped inhibit senescence.

### Overexpression of TZAP aggravated the loss of proliferation capacity and differentiation potential in pMSCs

**Fig. 4:**
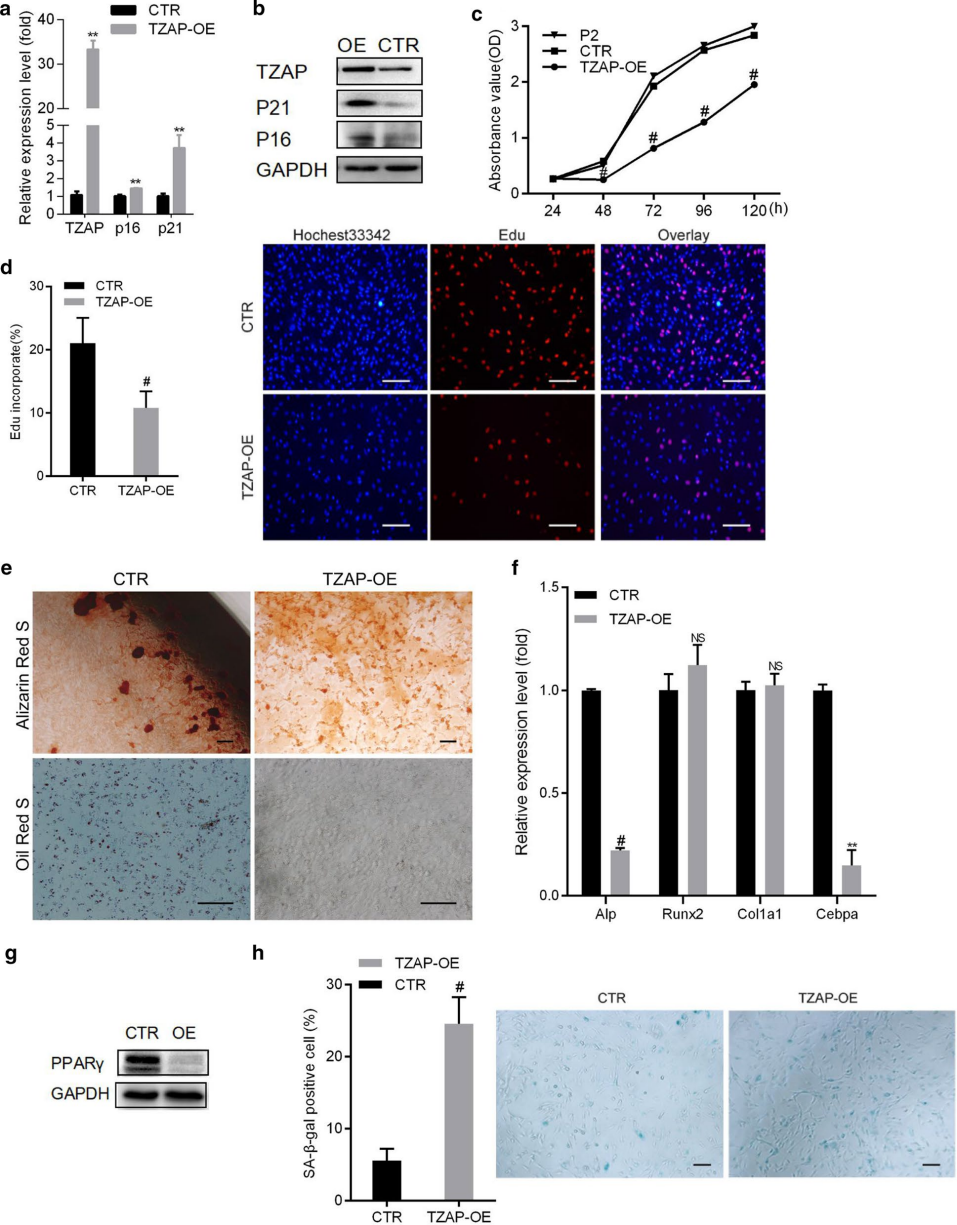
Overexpression of TZAP in early-passage MSCs decreased proliferation and differentiation potential. **a** The mRNA levels of TZAP and senescence-associated markers (*p21* and *p16*) in pMSCs were assessed by qRT-PCR after transduction with control plasmids (CTR) or TZAP overexpression vectors (TZAP-OE). **b** Protein levels of TZAP, P16INK14 (P16) and P21 in pMSCs were compared through western blot analysis after TZAP overexpression. **c** The effect of overexpression of TZAP on the cell growth rate. **d** Bar = 100 μm. The EdU incorporation rate of pMSCs with TZAP overexpression compared to that of the controls. **e** Bar = 100 μm. Differentiation of pMSCs with TZAP overexpression was assessed by oil red O staining after adipogenic induction or by alizarin red S staining after osteogenic induction. **f** The relevant expression levels of bone markers (*Alp*, *Runx2*, and *Col1a1*) and adipogenic markers (*Cebpa*) were assessed by qRT-PCR and compared between pMSCs with TZAP overexpression and the control cell line. **g** PPARγ expression was examined by western blotting upon TZAP overexpression in pMSCs. **h** Bar = 100 μm. SA-β-gal staining of pMSCs with TZAP overexpression compared to that of the control. All data are represented as the mean ± SEM. n = 3. *p < 0.05; **p < 0.01; ^#^*p *< 0.001, tested by one-way ANOVA

To further confirm the roles of TZAP in preventing senescence and maintaining stem-like properties, we also overexpressed TZAP in P2 pMSCs and evaluated its effect on proliferation capacity and lineage differentiation potential. First, the mRNA (Fig. 4a) and protein expression levels (Fig. 4b) of TZAP were upregulated in P2 pMSCs. The upregulation of TZAP significantly reduced the rate of cell growth (Fig. 4c) and the EdU incorporation rate (Fig. 4d) compared to those of pMSCs transduced with the control vector. Overexpression of TZAP in P2 pMSCs also decreased the osteogenic and adipogenic differentiation potential (Fig. 4e), which was validated by downregulation of the osteoblast-specific transcript *Alp* and adipocyte-related markers (*Cebpa* and PPARγ), respectively (Fig. 4f, g). Taken together, these results indicate that the overexpression of TZAP negatively impacted the differentiation potential and proliferation capacity of pMSCs, implicating TZAP in the downregulation of proliferation capacity and differentiation potential in pMSCs.

### Overexpression of TZAP aggravated premature senescence in pMSCs

Overexpression of TZAP in P2 pMSCs resulted in an increase in the percentage of SA-β-gal-positive cells by 17% (from 6 to 23%) (Fig. 4h). Western blot analysis showed that compared to the control vector, TZAP overexpression in pMSCs significantly increased the protein levels of P21 and P16 (Fig. 4b). Consistent with this finding, qRT-PCR also revealed upregulated transcript levels of *p21* and *p16Ink4a* (Fig. 4a). At early pMSC passages, senescence-associated markers were upregulated by TZAP overexpression, echoing the observation that TZAP expression in WT pMSCs increased as the cell passage number increased. Together, these data suggested that TZAP plays an essential role in regulating replicative senescence in pMSCs.

### TZAP knockout maintained the properties of pMSCs through the p53 pathway

**Fig. 5:**
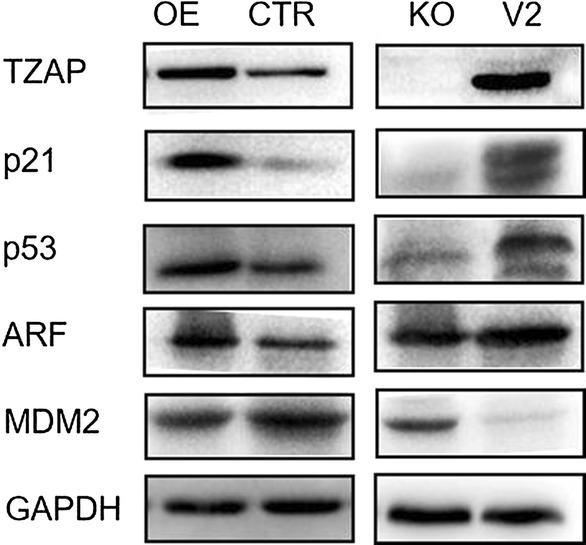
TZAP negatively regulates cell proliferation through the p53 pathway. Comparison of western blot detection of different intracellular protein levels (TZAP, P21, P53, ARF, and MDM2) in pMSCs with TZAP overexpression (OE) and TZAP knockout (KO)

TZAP has been reported to be involved in decreased cell proliferation activity by controlling the ARF-p53 pathway in HEK293T cells and primary HDFn cells (human dermal fibroblasts) [39]. It has also been demonstrated that ARF can significantly activate p53 through sequestering the transcription of MDM2, which then increases the expression of the p53 target gene p21 [40]. Accordingly, we investigated whether knockdown or overexpression of TZAP affects the expression of ARF in pMSCs. After overexpression of TZAP in pMSCs, western blot analysis showed that overexpression of TZAP led to increased levels of ARF, P53 and P21 but decreased levels of MDM2 compared to those in pMSCs transduced with the control vector (Fig. 5). In contrast, knockout of TZAP in pMSCs decreased ARF, P53, and P21 levels but increased MDM2 levels (Fig. 5). These data suggested that inhibition of TZAP improves proliferation capacity and differentiation potential and inhibits senescence via suppression of the p53 pathway in pMSCs.

## Discussion

As mentioned in the “Background” section, the wide range of clinical applications of MSCs requires large-scale culture expansion of MSCs in vitro, during which the cells exhibit senescence and a loss of multipotency after a few passages [38, 41]. We first demonstrated that the ablation of TZAP can maintain MSC proliferation and differentiation properties by repressing the expression of the cellular senescence markers p16INK4A and p21. TZAP is a POK family protein consisting of 11 zinc finger domains and a POZ domain, which is commonly rearranged in leukemia and leiomyoma or obliterated in various cancers, including melanoma, neuroblastoma, Merkel cell carcinomas, breast cancer, pheochromocytoma and colon carcinoma [42]. Previous studies have suggested that TZAP is a negative regulator of cell proliferation in cancer cells [31], but whether TZAP plays a role as a tumor suppressor remains uncertain. Recently, two groups reported that TZAP is a new specific telomere-binding protein that directly binds to telomeric TTAGGG repeats and stimulates telomere trimming [23, 43, 44]. However, how the TZAP protein functions in MSCs at the molecular, cellular, and genetic levels remains unknown. Our data showed that early-passage pMSCs exhibit better proliferation and multipotent differentiation capacity than late-passage pMSCs. Here, we first demonstrated that the mRNA and protein levels of TZAP were higher in late-passage pMSCs than in early-passage pMSCs. Most importantly, the increased expression of TZAP correlated well with the expression of the cellular senescence markers p16INK4A and p21. All these data suggest that high levels of TZAP are implicated in the progression of pMSC osteogenic/adipogenic differentiation and senescence.

To further explore whether the loss of TZAP affects pMSC properties, a knockout of TZAP was generated by CRISPR-Cas9 genome editing in pMSCs (Additional file 2: Fig. S1A–C). TZAP knockout in pMSCs increased the cell proliferation rate, promoted osteogenic and adipogenic differentiation potential and prevented premature senescence, which might indicate the ability of TZAP ablation to maintain proliferation and multipotent differentiation capacity. On the other hand, overexpression of TZAP reduced the cell proliferation rate, inhibited osteogenic and adipogenic differentiation potential and aggravated the aging of pMSCs. All these data suggest that low levels of TZAP expression prevent pMSCs from undergoing premature senescence and are beneficial for the cellular proliferation and osteogenic/adipogenic differentiation capacities of the cells.

It was previously reported that TZAP can negatively regulate cell proliferation by controlling the p53 pathway (ARF-MDM2-P53-CDKN1A/P21). TZAP activates the transcription of ARF by acting on the proximal promoter region (bp, − 149 ~ + 53) [39], which is upregulated during replicative senescence. ARF stabilizes p53 through forming a complex with MDM2 and catalyzing its degradation [45–47], thereby causing the cells to arrest in the G1 and G2 phases, resulting in cell cycle arrest or apoptosis [40, 48]. Our data showed that overexpression of TZAP induced the expression of ARF and p53 and repressed MDM2 in pMSCs. In contrast, knockout of TZAP in pMSCs decreased the expression of ARF. These findings indicate that TZAP may negatively regulate cell proliferation in pMSCs through affecting the p53 pathway. Taken together, we found that TZAP plays an inhibitory role in regulating and maintaining pMSC properties through the p53 pathway, which comprises ARF, MDM2, P53 and P21, and knockdown of TZAP protects the cells from premature senescence.

Our results suggest that early-passage pMSCs exhibit superior osteoblast differentiation and adipocyte cell differentiation potential compared to that of late-passage pMSCs. We found that knockout of TZAP in late-passage pMSCs significantly enhanced adipogenesis and osteogenic differentiation capacity in vitro, and overexpression of TZAP in early pMSCs led to decreased osteogenic and adipogenic differentiation potential in vitro. Furthermore, knockout of TZAP increased the expression of PPARγ and *Cebpa*, critical transcription factors for adipocyte differentiation, which led to an increase in adiposity in pMSCs [49, 50]. Interestingly, we found that knockout of TZAP may also increase the mRNA level of *Runx2* (a transcription factor that regulates osteogenic differentiation in MSCs [51]) and *Alp* (a widely known osteoblastic marker that has been used to evaluate bone formation capacity in osteoporosis [52]), while overexpression of TZAP in P2 pMSCs also led to downregulation of *Alp*, *Cebpa* and PPARγ. Collectively, our data indicated that knockout of TZAP facilitates maintenance of the differentiation capacity of pMSCs during senescence. These findings are relevant for the development of regenerative medicine protocols that use mesenchymal cells in cell-based therapy.

In summary, we isolated pMSCs from minipigs, which have physiological, anatomical and biochemical features comparable to those of humans, and then transfected the pMSCs with TZAP or knocked out TZAP to evaluate the multipotency of the genetically modified pMSCs in vitro. These data suggest that knockout of TZAP in pMSCs helps maintain MSC proliferation and multipotent differentiation properties and prevents the cells from undergoing senescence through the p53 pathway. However, a more comprehensive study is needed to explain the molecular mechanisms by which TZAP regulates the pluripotency of pMSCs. After sufficient evaluation, the molecular mechanism underlying the inhibitory role of TZAP in the maintenance of MSC stem-like properties may facilitate the design of new strategies for the clinical application of MSCs in cell therapy and tissue engineering.

## Conclusion

In conclusion, our study showed that the ablation of TZAP can improve pMSC proliferation and osteogenic/adipogenic differentiation properties and prevent senescence. Therefore, knockout or attenuation of intracellular TZAP could be a new strategy for genetically modifying pMSCs to improve the efficiency of mesenchymal cell therapy.

## Additional files

**Additional file 1: Fig. S1.** TZAP was knocked out in pMSCs by CRISPR/Cas9 gene editing. (A) A diagram of the porcine TZAP locus targeted by CRISPR editing and sequencing results for the edited porcine TZAP gene in the KO-sg2 clone. The sgRNA target region and PAM sequences are highlighted in cyan and red, respectively. (B and C) Pools of pMSCs treated with the control and pMSCs treated with Cas9 and TZAP-targeting sgRNA LV-sg2 were analyzed by TIDE [40]; (B) the spectra and frequency of Indels were determined by TIDE. (C) Visualization of the aberrant signal sequence in LV-sg2 (green) and the control sample (black), the region used for decomposition (gray bar) and the expected cutting site (blue dotted line).

**Additional file 2: Table S1.** Primers for qRT-PCR analysis of porcine TZAP and related gene expression.

## References

[1] Friedenstein AJ, Petrakova KV, Kurolesova AI, Frolova GP (1968). Heterotopic of bone marrow. Analysis of precursor cells for osteogenic and hematopoietic tissues. Transplantation.

[2] Friedenstein AJ, Chailakhjan RK, Lalykina KS (1970). The development of fibroblast colonies in monolayer cultures of guinea-pig bone marrow and spleen cells. Cell Tissue Kinet.

[3] Friedenstein AJ, Gorskaja JF, Kulagina NN (1976). Fibroblast precursors in normal and irradiated mouse hematopoietic organs. Exp Hematol.

[4] Castro-Malaspina H, Gay RE, Resnick G, Kapoor N, Meyers P, Chiarieri D, McKenzie S, Broxmeyer HE, Moore MA (1980). Characterization of human bone marrow fibroblast colony-forming cells (CFU-F) and their progeny. Blood.

[5] Keating A (2012). Mesenchymal stromal cells: new directions. Cell Stem Cell.

[6] Meirelles Lda S, Fontes AM, Covas DT, Caplan AI (2009). Mechanisms involved in the therapeutic properties of mesenchymal stem cells. Cytokine Growth Factor Rev.

[7] Caplan AI, Dennis JE (2006). Mesenchymal stem cells as trophic mediators. J Cell Biochem.

[8] Caplan AI (2017). Mesenchymal stem cells: time to change the name!. Stem Cells Transl Med.

[9] Squillaro T, Peluso G, Galderisi U (2016). Clinical trials with mesenchymal stem cells: an update. Cell Transplant.

[10] Chen YT, Sun CK, Lin YC, Chang LT, Chen YL, Tsai TH, Chung SY, Chua S, Kao YH, Yen CH (2011). Adipose-derived mesenchymal stem cell protects kidneys against ischemia-reperfusion injury through suppressing oxidative stress and inflammatory reaction. J Transl Med.

[11] Najar M, Krayem M, Merimi M, Burny A, Meuleman N, Bron D, Raicevic G, Lagneaux L (2018). Insights into inflammatory priming of mesenchymal stromal cells: functional biological impacts. Inflamm Res.

[12] Loebel C, Burdick JA (2018). Engineering stem and stromal cell therapies for musculoskeletal tissue repair. Cell Stem Cell.

[13] Campisi J, d’Adda di Fagagna F (2007). Cellular senescence: when bad things happen to good cells. Nat Rev Mol Cell Biol.

[14] Yu KR, Kang KS (2013). Aging-related genes in mesenchymal stem cells: a mini-review. Gerontology.

[15] Muraglia A, Cancedda R, Quarto R (2000). Clonal mesenchymal progenitors from human bone marrow differentiate in vitro according to a hierarchical model. J Cell Sci.

[16] Hayflick L (1965). The limited in vitro lifetime of human diploid cell strains. Exp Cell Res.

[17] Harley CB, Futcher AB, Greider CW (1990). Telomeres shorten during ageing of human fibroblasts. Nature.

[18] Bonab MM, Alimoghaddam K, Talebian F, Ghaffari SH, Ghavamzadeh A, Nikbin B (2006). Aging of mesenchymal stem cell in vitro. BMC Cell Biol.

[19] Baxter MA, Wynn RF, Jowitt SN, Wraith JE, Fairbairn LJ, Bellantuono I (2004). Study of telomere length reveals rapid aging of human marrow stromal cells following in vitro expansion. Stem Cells.

[20] Sobinoff AP, Pickett HA (2017). Alternative lengthening of telomeres: DNA repair pathways converge. Trends Genet.

[21] d’Adda di Fagagna F, Reaper PM, Clay-Farrace L, Fiegler H, Carr P, Von Zglinicki T, Saretzki G, Carter NP, Jackson SP (2003). A DNA damage checkpoint response in telomere-initiated senescence. Nature.

[22] Raz V, Vermolen BJ, Garini Y, Onderwater JJ, Mommaas-Kienhuis MA, Koster AJ, Young IT, Tanke H, Dirks RW (2008). The nuclear lamina promotes telomere aggregation and centromere peripheral localization during senescence of human mesenchymal stem cells. J Cell Sci.

[23] Li JS, Miralles Fuste J, Simavorian T, Bartocci C, Tsai J, Karlseder J, Lazzerini Denchi E (2017). TZAP: a telomere-associated protein involved in telomere length control. Science.

[24] Krishnamurthy J, Torrice C, Ramsey MR, Kovalev GI, Al-Regaiey K, Su L, Sharpless NE (2004). Ink4a/Arf expression is a biomarker of aging. J Clin Invest.

[25] Kim WY, Sharpless NE (2006). The regulation of INK4/ARF in cancer and aging. Cell.

[26] Gil J, Peters G (2006). Regulation of the INK4b-ARF-INK4a tumour suppressor locus: all for one or one for all. Nat Rev Mol Cell Biol.

[27] Carrasco-Garcia E, Moreno M, Moreno-Cugnon L, Matheu A (2017). Increased Arf/p53 activity in stem cells, aging and cancer. Aging Cell.

[28] Bracken AP, Kleine-Kohlbrecher D, Dietrich N, Pasini D, Gargiulo G, Beekman C, Theilgaard-Monch K, Minucci S, Porse BT, Marine JC (2007). The Polycomb group proteins bind throughout the INK4A-ARF locus and are disassociated in senescent cells. Genes Dev.

[29] Sohn M, Korn V, Imanidis G (2015). Porcine ear skin as a biological substrate for in vitro testing of sunscreen performance. Skin Pharmacol Physiol.

[30] Stiefel C, Schwack W (2014). Reactivity of cosmetic UV filters towards skin proteins: model studies with Boc-lysine, Boc-Gly-Phe-Gly-Lys-OH, BSA and gelatin. Int J Cosmet Sci.

[31] Sheu SY, Wang WL, Fu YT, Lin SC, Lei YC, Liao JH, Tang NY, Kuo TF, Yao CH (2014). The pig as an experimental model for mid-dermal burns research. Burns.

[32] Held M, Rothenberger J, Schiefer J, Rath R, Petersen W, Jaminet P, Schaller HE, Rahmanian-Schwarz A (2014). Alteration of biomechanical properties of skin in acute cold contact injury. Burns.

[33] Lee WJ, Park JS, Jang SJ, Lee SC, Lee H, Lee JH, Rho GJ, Lee SL (2016). Isolation and cellular phenotyping of mesenchymal stem cells derived from synovial fluid and bone marrow of minipigs. J Vis Exp.

[34] Soleimani M, Nadri S (2009). A protocol for isolation and culture of mesenchymal stem cells from mouse bone marrow. Nat Protoc.

[35] Sanjana NE, Shalem O, Zhang F (2014). Improved vectors and genome-wide libraries for CRISPR screening. Nat Methods.

[36] Brinkman EK, Chen T, Amendola M, van Steensel B (2014). Easy quantitative assessment of genome editing by sequence trace decomposition. Nucleic Acids Res.

[37] Zhu L, Zhang X, Fu X, Li Z, Sun Z, Wu J, Wang X, Wang F, Li X, Niu S (2018). TIPE2 suppresses progression and tumorigenesis of esophageal carcinoma via inhibition of the Wnt/beta-catenin pathway. J Transl Med.

[38] Yew TL, Chiu FY, Tsai CC, Chen HL, Lee WP, Chen YJ, Chang MC, Hung SC (2011). Knockdown of p21(Cip1/Waf1) enhances proliferation, the expression of stemness markers, and osteogenic potential in human mesenchymal stem cells. Aging Cell.

[39] Yoon JH, Choi WI, Jeon BN, Koh DI, Kim MK, Kim MH, Kim J, Hur SS, Kim KS, Hur MW (2014). Human Kruppel-related 3 (HKR3) is a novel transcription activator of alternate reading frame (ARF) gene. J Biol Chem.

[40] Stott FJ, Bates S, James MC, McConnell BB, Starborg M, Brookes S, Palmero I, Ryan K, Hara E, Vousden KH, Peters G (1998). The alternative product from the human CDKN2A locus, p14(ARF), participates in a regulatory feedback loop with p53 and MDM2. EMBO J.

[41] Shibata KR, Aoyama T, Shima Y, Fukiage K, Otsuka S, Furu M, Kohno Y, Ito K, Fujibayashi S, Neo M (2007). Expression of the p16INK4A gene is associated closely with senescence of human mesenchymal stem cells and is potentially silenced by DNA methylation during in vitro expansion. Stem Cells.

[42] Maris JM, Jensen J, Sulman EP, Beltinger CP, Allen C, Biegel JA, Brodeur GM, White PS (1997). Human Kruppel-related 3 (HKR3): a candidate for the 1p36 neuroblastoma tumour suppressor gene?. Eur J Cancer.

[43] Jahn A, Rane G, Paszkowski-Rogacz M, Sayols S, Bluhm A, Han CT, Draskovic I, Londono-Vallejo JA, Kumar AP, Buchholz F (2017). ZBTB48 is both a vertebrate telomere-binding protein and a transcriptional activator. EMBO Rep.

[44] Zhao Y, Zhang G, He C, Mei Y, Shi Y, Li F (2018). The 11th C2H2 zinc finger and an adjacent C-terminal arm are responsible for TZAP recognition of telomeric DNA. Cell Res.

[45] Weber JD, Taylor LJ, Roussel MF, Sherr CJ, Bar-Sagi D (1999). Nucleolar Arf sequesters Mdm2 and activates p53. Nat Cell Biol.

[46] Moll UM, Petrenko O (2003). The MDM2-p53 interaction. Mol Cancer Res.

[47] Juven-Gershon T, Oren M (1999). Mdm2: the ups and downs. Mol Med.

[48] Silva J, Silva JM, Dominguez G, Garcia JM, Cantos B, Rodriguez R, Larrondo FJ, Provencio M, Espana P, Bonilla F (2003). Concomitant expression of p16INK4a and p14ARF in primary breast cancer and analysis of inactivation mechanisms. J Pathol.

[49] Luz-Crawford P, Ipseiz N, Espinosa-Carrasco G, Caicedo A, Tejedor G, Toupet K, Loriau J, Scholtysek C, Stoll C, Khoury M (2016). PPARbeta/delta directs the therapeutic potential of mesenchymal stem cells in arthritis. Ann Rheum Dis.

[50] Richard AJ, Stephens JM (2011). Emerging roles of JAK-STAT signaling pathways in adipocytes. Trends Endocrinol Metab.

[51] Chen Q, Shou P, Zheng C, Jiang M, Cao G, Yang Q, Cao J, Xie N, Velletri T, Zhang X (2016). Fate decision of mesenchymal stem cells: adipocytes or osteoblasts?. Cell Death Differ.

[52] Garnero P (2008). Biomarkers for osteoporosis management: utility in diagnosis, fracture risk prediction and therapy monitoring. Mol Diagn Ther.

