# Supplementary material for: TZAP plays an inhibitory role in the self-renewal of porcine mesenchymal stromal cells and is implicated the regulation of premature senescence via the p53 pathway
Source: J Transl Med. 2019 Mar 7;17:72. doi: 10.1186/s12967-019-1820-8 (PMC6404308; doi:10.1186/s12967-019-1820-8)
Supplement: Supplementary file 1 — Additional file 1: Fig. S1. TZAP was knocked out in pMSCs by CRISPR/Cas9 gene editing. (A) A diagram of the porcine TZAP locus targeted by CRISPR editing and sequencing results for the edited porcine TZAP gene in the KO-sg2 clone. The sgRNA target region and PAM sequences are highlighted in cyan and red, respectively. (B and C) Pools of pMSCs treated with the control and pMSCs treated with Cas9 and TZAP-targeting sgRNA LV-sg2 were analyzed by TIDE [40]; (B) the spectra and frequency of Indels were determined by TIDE. (C) Visualization of the aberrant signal sequence in LV-sg2 (green) and the control sample (black), the region used for decomposition (gray bar) and the expected cutting site (blue dotted line). [file 12967_2019_1820_MOESM1_ESM.docx]

**Additional Table S1 Primers for qRT-PCR analysis of porcine TZAP and the related gene expression**

| **Gene** | **Forward Primer (5’-3’)** | **Reverse Primer (5’-3’)** |
| --- | --- | --- |
| pGAPDH | GGTGATGCTGGTGCTGAGT | ACAGTCTTCTGGGTGGCAGT |
| pTZAP | CTCGGAACAGGTCTTCAC | AGGAGGAGCACTTGTAGG |
| p21 | ACATAGGGTTCCCCAGTT | GTAGTTCCAGGCGTTGATT |
| P16INK4A | GAATATGGTGCGCCGTCTCT | TCCTCACTAGCAACAGCACG |
| ALP | ACCACCACGAGAGTGAACCA | CGTTGTCTGAGTACCAGTCCC |
| RUNX2 | CCGCCTCAGTGATTTAGGGC | GGGTCTGTAATCTGACTCTGTCC |
| COL1A1 | AGGGCCAAGACGAAGACATC | CAGATCACGTCATCGCACAAC |
| CEBPA | TGGACAAGAACAGCAACGAG | TCACTGGTCAACTCCAGCAC |
