# Supplementary figures and images for: TZAP plays an inhibitory role in the self-renewal of porcine mesenchymal stromal cells and is implicated the regulation of premature senescence via the p53 pathway
Source: J Transl Med. 2019 Mar 7;17:72. doi: 10.1186/s12967-019-1820-8 (PMC6404308; doi:10.1186/s12967-019-1820-8)

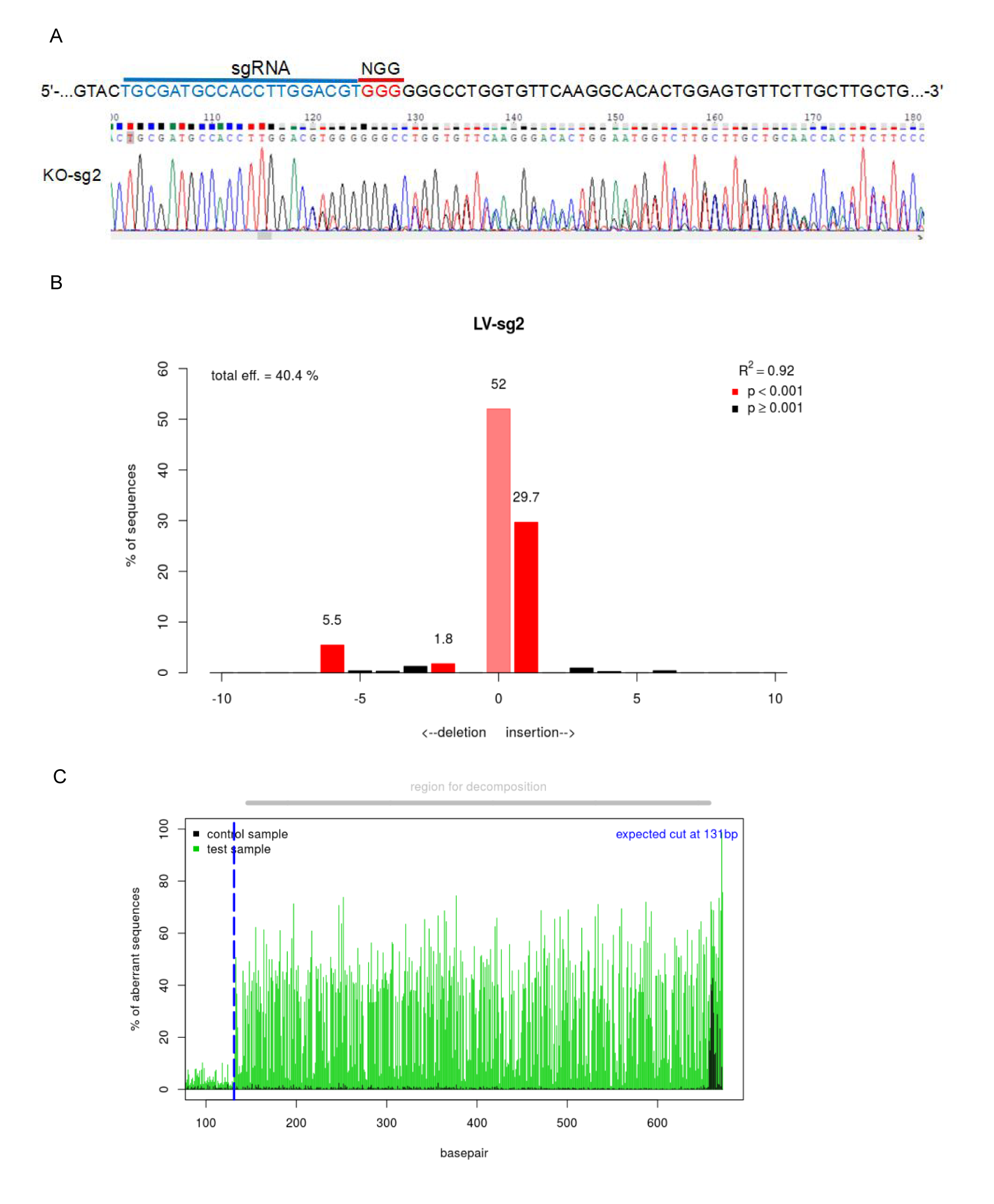

Supplement: Supplementary file 2 — Additional file 2: Table S1. Primers for qRT-PCR analysis of porcine TZAP and related gene expression. [file 12967_2019_1820_MOESM2_ESM.tif]
